# Editorial: Mental health: cell models to mechanisms

**DOI:** 10.3389/fcell.2023.1244425

**Published:** 2023-07-25

**Authors:** Adrian J. Harwood, Spyros Petrakis, Yavuz Oktay, R. Jeroen Pasterkamp

**Affiliations:** ^1^ Neuroscience and Mental Health Innovation Institute (NMHII), Cardiff University, Cardiff, United Kingdom; ^2^ Centre for Research and Technology Hellas (CERTH), Thessaloniki, Greece; ^3^ Izmir International Biomedicine and Genome Institute, Dokuz Eylül University, Izmir, Türkiye; ^4^ Department of Translational Neuroscience, UMC Utrecht Brain Center, UMC Utrecht, Utrecht University, Utrecht, Netherlands

**Keywords:** mental health, cell biology, IPSC, neurodevelopment, *in vitro* model

A significant barrier to our understanding of mental health, and subsequent development of improved preventative and therapeutic strategies, is a fundamental lack of molecular mechanistic knowledge. This Research Topic focuses on the use of *in vitro* cell studies to understand the biology of mental health. In this context, human stem cell technology, in particular the application of induced pluripotent stem cells (iPSCs), is poised to open a previously unimagined analytical capability. The key to maximize the benefits of this new opportunity will be the design of effective patient-derived, cell-based assays and their analysis. Here, we bring together a selection of research articles and technical perspectives that exemplify this approach.

The human brain is a complex, large-scale organ that changes throughout the lifespan. The differences between individual people arise through microstructural cell interactions within the brain and how they modulate neuronal function. The objective of *in vitro* cell assays is therefore not to faithfully reconstruct the whole brain, this can be achieved by post-mortem brain histology, functional brain imaging and animal studies, but rather to probe these interactions at high resolution and in manipulable cell culture conditions. However, to achieve this, it is necessary to make clear decisions on what biological processes to study.

The most immediate benefit of an *in vitro* cell culture system is the ability to grow single cell types and study their cell morphology, RNA and protein composition and their response to addition of exogeneous molecules in their microenvironment, such as cell signal molecules, dietary components, or potential therapeutics. The utility of such experimental designs has been substantially amplified by our ability to generate and expand human neuronal and glial cell types to significant quantities. An important feature often not appreciated is that *in vitro* neurodevelopment allows the experimenter to follow the progression of cell differentiation, and such studies have shown that the cells derived from patients diagnosed with a Neurodevelopmental Disorder (NDD) often exhibit altered *in vitro* neurodevelopmental timing.

Currently, 2D *in vitro* cell culture systems remain the main workhorse employed for most studies, including a number of those reported in this Research Topic. Due to their adherence on a flat surface, cultures are easy to maintain, are very suitable for high-resolution light-based imaging and when plated on Multielectrode Arrays (MEAs) can measure ensemble of synapse-connected networks of neurons and emergent oscillatory pattens of neuronal activity. This simple plate-based format can be up-scaled by use of multi-well plates for high throughput assays and hence platforms for drug screening and target validation.

Cells within the brain, however, develop and function in very different environments to those in 2D cell culture. They interact with a diverse array of different cell types and extracellular components; their developmental programmes are tuned by the activity of other cells around them; and restrictions to diffusion dramatically can change exposure to signal molecule and metabolites in comparison to cells in culture. To close this gap more complex 3D multicellular systems, such as brain organoids, can be employed that develop multiple cell neuronal and glial cell types *in situ*. Techniques for their generation and analysis are advancing all the time, although challenges remain such size variation, cell viability and neuronal functional monitoring by MEA in a 3D culture. The ambition is to establish methods for eliciting and measuring more complex brain-like behaviours.

A parallel powerful technology with a major impact for *in vitro* cell studies is transcriptional profiling by RNAseq, and related “omics” methods to measure gene transcription activity and its epigenetic modification. When carried out at a high read-depth and a genome-wide scale Differentially Expressed Gene (DEG) analysis offers a discovery tool to identify mental health-related genes. However, at lower read-depth, RNAseq delivers an effective method for tracking neurodevelopmental progress. In single cell mode, RNAseq can be effective for profiling cell types in 2D and 3D mixed cell cultures. As technologies capable of delivering cell and subcellular resolution *in situ*, multiplexed transcription profiling (Spatial Biology) become mainstream, in the future the modelling approaches in this theme will further enable cell type proportion and interaction measurements in organoid and high-density cultures.

None of these advanced and complex cell-based methodologies are effective without a substantial associated computational analysis. Different types of expression-based, high-content sequence data can be overlaid to indicate potential underlying mechanism. This further needs to be integrated with cell type, function, and development. At the higher-scale of multicellular organisation cell-cell interactions, including synaptic and neuronal connectivity, need to be identified, modelled *in silico* and tested *in vitro*. In combination, capacity for multiscale integration of *in vitro* cell data provides a powerful research capability to study and experimentally manipulate neurodevelopment and function, particularly for human-based studies where studies on primary tissue are often very challenging.

Finally, to be truly beneficial, *in vitro* cell studies need to reside in a greater landscape of other approaches to patient and animal-based studies; the need to refine, not replace. An ideal *in vitro* cell model should provide unique neurobiological insights, but easily align this new knowledge with equivalent biological measures from patient and animal studies. Such translatability of *in vitro* cell mechanism will drive a revolution in our understanding and capacity to deliver therapies for mental health.

In summary, this Research Topic aims to exemplify key considerations for good design and analysis of *in vitro* cell studies. This will drive a new cell and development understanding of brain biology and mental health.

